# Genetic testing results of children suspected to have Stickler syndrome type collagenopathy after ocular examination

**DOI:** 10.1002/mgg3.1628

**Published:** 2021-05-05

**Authors:** Arif O. Khan, Lama AlAbdi, Nisha Patel, Rana Helaby, Mais Hashem, Firdous Abdulwahab, Fahad B. AlBadr, Fowzan S. Alkuraya

**Affiliations:** ^1^ Department of Genetics KFSHRC Riyadh Saudi Arabia; ^2^ Eye Institute Cleveland Clinic Abu Dhabi Abu Dhabi United Arab Emirates; ^3^ Department of Ophthalmology Cleveland Clinic Lerner College of Medicine of Case Western University Cleveland OH USA; ^4^ College of Science King Saud University Riyadh Saudi Arabia; ^5^ Department of Radiology College of Medicine King Saud University Riyadh Saudi Arabia; ^6^ Department of Anatomy and Cell Biology College of Medicine Alfaisal University Riyadh Saudi Arabia

**Keywords:** cataract, *COL11A1*, *COL2A1*, *COL9A1*, Stickler syndrome, vitreous

## Abstract

**Purpose:**

Stickler syndrome is a collagenopathy that is typically *COL2A1*‐related (autosomal dominant) and less commonly related to other collagen gene mutations. Diagnosis is straightforward when a child has myopia or retinal detachment in the setting of classic diagnostic criteria such as hearing impairment, midfacial hypoplasia, and arthropathy. However, some children have primarily ocular disease with mild or no extraocular features. Such children can remain undiagnosed unless suspicion is raised by the ophthalmologist.

**Methods:**

Retrospective consecutive case series (2014–2016) of children (<12 years old) suspected to have Stickler syndrome type collagenopathy by a single ophthalmologist and able to complete genetic testing for this possibility. Suspicion was based on vitreous abnormalities and myopia or lens opacities in the setting of prior retinal detachment, hearing impairment, or facial flatness.

**Results:**

Average age of the 12 identified children was 8 years old (range 3–11; five boys). Average spherical equivalent for phakic eyes was −13 (range −3.5 to −30). Nine children had lens opacities or aphakia; two with aphakia also had lens subluxation or iridodonesis. Other recurrent clinical features included flat facies (12/12), hearing impairment (5/12), and prior retinal detachment (4/12). Pathogenic variants for collagenopathy were uncovered in 10/12 children: *COL11A1* (heterozygous) in six, *COL2A1* (heterozygous) in two, and *COL9A1* (homozygous) in two. One child was homozygous for pathogenic variation in *LRPAP1*. One child had no detectable gene mutations.

**Conclusions:**

Taken together, these clinical features (particularly vitreous abnormality, myopia, and lens opacity) had a high molecular yield for collagen gene mutation. Ophthalmologists who see such children should suspect Stickler syndrome, even in the absence of overt systemic disease. *COL11A1*‐related rather than *COL2A1*‐related autosomal dominant disease may be more common when undiagnosed children are identified based on ocular examination. Biallelic mutations in *LRPAP1* can result in a phenotype that may resemble Stickler syndrome.

## Graphical abstract

In this study we report our experience with gene mutations in children suspected to have Stickler syndrome type collagenopathy based on ocular examination.To the best of our knowledge this is the first such study based on ascertainment by an ophthalmologist.

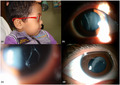

## 1 INTRODUCTION

Stickler syndrome (hereditary progressive arthro‐ophthalmopathy) is a variable collagenopathy phenotype with characteristic auditory, oro‐facial, skeletal, and ocular features (Rose et al., 2005; Snead & Yates, 1999). Hearing impairment ranges from none to severe. Midface hypoplasia can manifest as depressed nasal bridge, maxillary hypoplasia, micrognathia, or flatness to the face. Additional features can include bifid uvula, cleft palate, or Pierre Robin sequence. Potential skeletal findings include juvenile osteoarthritis, early adult‐onset degenerative joint disease, or mild spondyloepiphyseal dysplasia. Ocular findings include pediatric high myopia, lens opacities, vitreous changes, and predisposition to retinal detachment. Globally, the most common form of Stickler syndrome is autosomal dominant *COL2A1*‐related Sticker syndrome Type 1 (Robin et al., 2017; Rose et al., 2005; Snead & Yates, 1999), which accounts for 80%–90% of cases (Robin et al., 2017). *COL2A1* (OMIM 120140) encodes the three identical alpha‐1 chains that comprise collagen type II, the major component of vitreous collagen fibrils. Diagnostic probability for Stickler Syndrome Type 1 can be clinically scored using a points system based on involvement criteria of auditory, oro‐facial, skeletal, and ocular systems (Rose et al., 2005). However, some patients with *COL2A1* mutation have minimal or no extraocular involvement (Richards et al., 2000; Rose et al., 2005; Snead et al., 2011).

In addition to collagen type II (75% of vitreous collagen), other components of vitreous collagen fibrils are collagen types V/XI (<10% of vitreous collagen) and IX (25% of vitreous collagen) (Le Goff & Bishop, 2008). Mutations in collagen types XI and IX are less common causes of Stickler syndrome. Collagen type V/XI, located close to the surface of vitreous collagen fibrils, is unique to the vitreous in that its triple helical molecule contains alpha chains encoded by genes for both collagen type V and collagen type XI. Heterozygous mutations in *COL11A1* (OMIM 120280) or *COL11A2* (OMIM 120290) have been associated with autosomal dominant disease, although *COL11A2*‐related disease lacks ophthalmic findings as the gene is not expressed in the vitreous (Le Goff & Bishop, 2008). *COL11A1*‐related disease accounts for 10%–20% of Stickler syndrome (Robin et al., 2017). Collagen type IX, located on the surface of the collagen fibrils, is a heterotrimer of disulfide bonded alpha‐1, alpha‐2, and alpha‐3 chains, each encoded by *COL9A1* (OMIM 120210), *COL9A2* (OMIM 120260), and *COL9A3* (OMIM 120270), respectively. Biallelic mutations in *COL9A1*, *COL9A2*, and *COL9A3* are rare causes of autosomal recessive disease (Nixon et al., 2019; Robin et al., 2017).

Most genetic studies of Stickler syndrome describe patients identified based on multisystem involvement and, thus, have a high diagnostic probability score. Children with minimal or no extraocular involvement are less well described and can remain undiagnosed unless an ophthalmologist raises the possibility (Rose et al., 2005). In this study, we report our experience with genetic testing for children suspected to have Stickler syndrome type collagenopathy based on ocular examination.

## 2 METHODS

### 2.1 Ethical compliance

The study adhered to the Declaration of Helsinki and had institutional board review approval.

This case series is comprised of a cohort of Arab children (<12 years old) who were suspected to have Stickler syndrome type collagenopathy by a single ophthalmologist (AOK) and were able to complete genetic analysis for this possibility. The suspicion was based on the clinician's experience––minimum criteria were vitreous abnormalities and myopia or lens opacities in the setting of one or more of the following: prior retinal detachment, hearing impairment, facial flatness (considered a soft sign). Vitreous changes were graded as membranous (folded membrane), beaded (string‐like with beads), or nonspecific (wispy). Children diagnosed with or suspected of Stickler syndrome prior to ophthalmology referral were excluded. Also excluded were children whose clinical examination suggested other recognizable phenotypes of pediatric myopia (e.g., early‐onset glaucoma (Khan, 2011), retinal dystrophies (Hendriks et al., 2017), Knobloch syndrome (Khan et al., 2012), Donnai–Barrow syndrome (Khan & Ghazi, 2018), *LEPREL1*‐related myopia (Khan et al., 2015), and *LRPAP1*‐related myopia (Khan et al., 2016).

For genetic analysis, most patients underwent diagnostic Sanger sequencing of *COL2A1*. If the result was negative or if this was not done, then, informed consent was obtained under an institutional review board approved protocol for genetic analysis of causes of visual impairment (KFSHRC RAC# 2070023). Venous blood was collected in EDTA tubes for DNA extraction and downstream analyses. We utilized a multigene panel (Saudi Mendeliome Group, 2015) that captures and sequences the coding/splicing regions of 322 genes which have been previously recognized to cause human eye disease, including variants established to cause autosomal dominant or recessive Stickler syndrome. Whole‐exome sequencing was performed on a subset of cases as previously describe (Saudi Mendeliome Group, 2015). Variant classification followed the recommendations of the American College of Medical Genetics (Richards et al., 2015). Sanger sequencing of an identified candidate gene was done for the proband. Whenever possible, confirmatory segregation analysis was completed for all available family members.

## 3 RESULTS

**FIGURE 1:**
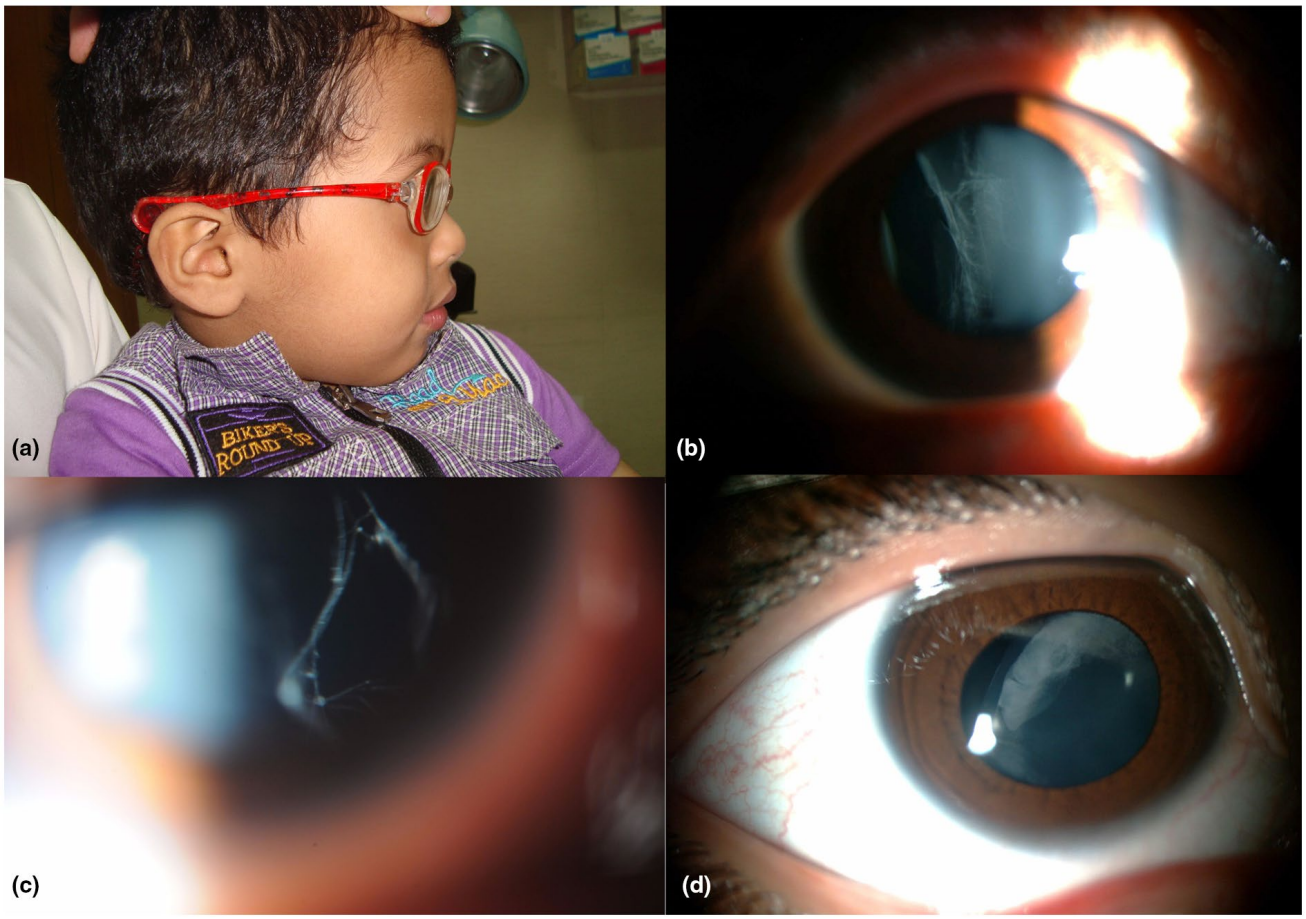
(Clinical examples): (a) flat face in side profile (subject #6); (b) right eye membranous vitreous abnormality (subject #1); (c) right eye beaded vitreous abnormality (subject #7); (d) right eye lens subluxation and peripheral lens opacities (subject #5)

Twelve Arab children (11 families) met the inclusion criteria. Ophthalmology referral was for strabismus or myopia. Average age of the 12 children was 8 years old (range 3–11; five boys). Average spherical equivalent for phakic eyes was −13 (range −3.5 to −30.0). Nine children had lens opacities or aphakia. Two with lens opacities also had lens subluxation (coronal displacement) or iridodonesis (vibration of the iris with eye movement), indicative of zonular weakness. Other recurrent clinical features included flat facies (12/12), hearing impairment (5/12), and prior retinal detachment (4/12) [Table 1]. Clinical examples are provided in Figure 1. Pathogenic variants for collagenopathy were uncovered in 10/12 children: *COL11A1* (heterozygous) in six, *COL2A1* (heterozygous) in two, and *COL9A1* (homozygous) in two. [Table 2, Table S1]. Regarding the other two children, one was homozygous for pathogenic variation in *LRPAP1* (OMIM 104225) and the other had no detectable gene mutations [Table 2]. As a subgroup, the 10 children with collagen gene mutations (subjects #1–10) had the same age average and range as the cohort as a whole and a similar phakic eye spherical equivalent mean (−12, range −3.5 to −20.0).

**TABLE 1:** Clinical features

#	Age	Sex	Diff hear	Prior RD	Myopia	BCVA	Lens	Vitreous	Comments
1	6 years	F	Yes	OS	−11	20/40	Clear	Membranous	Moderate exotropia OS
					N/A	LP	Total cat	Membranous	
2	8 years	M	Yes	OD	N/A	LP	Total cat	N/A	Cleft palate repair; bad eczema
					−11	20/20	Clear	Membranous	
3	8 years	F	Yes	No	−12	20/50	Clear	Wispy	—
					−11.50	20/50	Clear	Wispy	
4	7 years	F	Yes	No	N/A	20/30	Aphakic	N/A	Lensectomy for trauma OD; bowed knees
					−9	20/20	Clear	Wispy	
5	11 years	F	No	No	−18	20/70	Sup cat	Beaded	Lens subluxation OD; large esotropia; OS retinal detachment at age 13y
					−16	20/200	Sup cat	Beaded	
6	3 years	M	No	No	−20	Follows	Clear	Beaded	Small esotropia
					−18	Follows	Clear	Beaded	
7	11 years	M	No	OS	−11	20/50	Inf cat	Beaded	High arched palate, small uvula
					N/A	LP	Total cat	N/A	
8	4 years	M	No	No	−14	20/80	Clear	N/A	Very large esotropia
					−14	20/50	Clear	N/A	
9	7 years	F	Yes	No	−12	20/200	Sup cat	Beaded	Sibling of #10
					−6.50	20/60	Sup cat	Beaded	
10	10 years	F	No	No	−3.50	20/80	Post cat	N/A	Sibling of #9
					−3.75	20/40	Clear	Beaded	
11	5 years	F	No	OS	−30	20/200	Clear	Wispy	Lensectomy OS related to prior RD surgery
					N/A	LP	Aphakia	N/A	
12	11 years	M	No	No	−17	20/200	Inf cat	Wispy	Small esotropia; iridodonesis OU
					−18	20/70	Inf cat	Wispy	

Note:

Where two rows are present for an entry, upper row is right eye and lower row is left eye. All had a flat facies.

Abbreviations: BCVA, best‐corrected visual acuity; cat, cataract; DIFF HEAR, difficulty hearing by history; F, female; inf, inferior; M, male; N/A, not applicable or able; OD, right eye; OS, left eye; OU, both eyes; RD, retinal detachment; sup, superior.

**TABLE 2:** Genetic results

#	Gene	Pathogenic variant [zygosity]	Parents
1	*COL2A1*	NM_001844.5:c.2659C>T; (p.Arg887Ter) [heterozygous]	Same tribe
2	*COL2A1*	NM_001844.4:c.2818C>T; p.(Arg940*)[heterozygous]	No relation
3	*COL9A1*	NM_001851.4:c.1052C>A; p.(Ser351*) [homozygous]	Cousins
4	*COL9A1*	NM_078485.4:c.1339_1340del; p.(Arg447Glyfs*17) [homozygous]	Cousins
5	*COL11A1*	NM_080630.4:c.3408_3414del; p.(Glu1137Valfs*17) [heterozygous]	Same tribe
6	*COL11A1*	NM_001854.3:c.1945‐1G>C [heterozygous]	Cousins
7	*COL11A1*	NM_001854.3:c.2241+5G>T [heterozygous]	Cousins
8	*COL11A1*	NM_001854.4:c.3816+1G>A [heterozygous]	Cousins
9	*COL11A1*	NM_080630.4:c.4064G>A; p.(Gly1355Asp)[heterozygous]	Cousins
10			
11	*LRPAP1*	NM_002337.3:c.863_864del; p.(Ile288Argfs*118) [homozygous]	Cousins
12	None	None identified	Cousins

## 4 DISCUSSION

Taken together, these clinical features (particularly vitreous abnormality, myopia, and lens opacity) had a high molecular yield for collagen gene mutation. Based on our series, ophthalmologists who see such children should suspect Stickler syndrome type collagenopathy and consider genetic testing for definitive diagnosis. *COL11A1*‐related rather than *COL2A1*‐related autosomal dominant disease may be more common when undiagnosed children are identified based on ocular exam. Biallelic mutations in *LRPAP1* can result in a phenotype that may resemble Stickler syndrome.

Myopia, vitreous changes, and lens opacities were the major ocular findings in this cohort. The myopia in Stickler syndrome is typically early‐onset (often congenital), high (typically more myopia than −4), relatively stable, and associated with risk for retinal detachment (Wilson et al., 1996). However, lower degrees of myopia and even hyperopia are possible (Wilson et al., 1996). Such patients are often not included in published series unless they are relatives of a proband or have frank extraocular findings because retinal detachment or high myopia is often a criterion for diagnosis. One child in our cohort (subject #10) had low myopia (spherical equivalent of −3.5) but was the sibling of a proband initially referred for high myopia (subject #9) who was found to have collagenopathy. Slit‐lamp examination of subject #10 with attention to the vitreous revealed a beaded abnormality (as was found in his brother, subject #9) and genetic testing confirmed a heterozygous *COL11A1* mutation in both siblings. Beaded and membranous vitreous abnormalities have been described as predictive for mutation in *COL21A* or *COL11A1*, respectfully, while nonspecific vitreous abnormalities do not correlate with specific gene mutation (Snead et al., 2011; Snead & Yates, 1999). This was our experience in the current series. Lens opacities independent of retinal detachment have been described in up to 50% of patients with Stickler syndrome, depending upon patient age, and are often peripheral or sectoral (Wilson et al., 1996). Lens opacity or aphakia was present in the majority of our cases (9/12 in the entire cohort, 7/10 in those with collagen gene mutation), and all were pediatric cases as per our methodology. This high percentage of lens abnormalities is likely related to how these children were ascertained, that is, not by multisystemic diagnostic criteria but rather by an ophthalmologist. Zonular weakness, as evidenced by lens subluxation or iridodonesis, is a less common finding (Spallone, 1987). One child with collagen gene mutation had lens subluxation (subject #5). The one child for whom no gene mutation was identified had iridodonesis (subject #12).

Flat facies, hearing impairment, and prior retinal detachment were other recurrent clinical features in this cohort. Flat facies is a subjective judgment regarding the absence of concavity or convexity in side profile (Allanson et al., 2009). It can be present in normal individuals and is not present in all patients with Stickler syndrome type collagenopathy. In this series, it was not only evident in all children with underlying collagen gene mutation, but was also noted in the two children who did not have collagen gene mutations (subjects #11, 12). Hearing impairment is a classic diagnostic criterion for Stickler syndrome (Robin et al., 2017; Rose et al., 2005). Hearing impairment was noted in five children, all of whom had a collagen gene mutation. However, although all in this series were specifically questioned regarding hearing impairment because they were suspected of Stickler syndrome type collagenopathy, there was no formal testing. Audiologic examination may have uncovered subclinical hearing impairment in some. In addition, some may have had hearing impairment for reasons unrelated to their collagenopathy. Retinal detachment is also a classic diagnostic criterion for Stickler syndrome (Robin et al., 2017; Rose et al., 2005). The risk for retinal detachment tends to correlate with the degree of myopia and age of the patient, but retinal detachment can still occur in the context of lower refractive errors and in young children. Three of the 10 children with collagen gene mutations had prior retinal detachment in one eye (subjects #1, 2, 7). Although only a minority of children with collagenopathy had prior retinal detachment at the time of this study, all remain at risk for the complication as they get older. Subject #5 is known to have developed retinal detachment after data collection was complete for this study. Pediatric retinal detachment is unusual in general and should raise suspicion for Stickler syndrome in children even if they lack other classic diagnostic criteria. However, there are other rarer causes for pediatric retinal detachment, such as biallelic *LRPAP1* mutations, the underlying genotype for subject #11 (discussed further below). The occurrence of retinal detachment is an independent risk factor for cataract.

*COL11A1*‐related autosomal dominant disease was most common in our series, followed by *COL2A1*‐related autosomal dominant disease. However, *COL11A1*‐related disease accounts for only 10–20% of Stickler syndrome worldwide while *COL2A1*‐related disease accounts for 80–90% of cases (Robin et al., 2017). Again, this is likely related to our method of ascertainment, which was by an ocular examination. Prior genetic studies of Stickler syndrome are cohorts of patients diagnosed based on systemic diagnostic criteria. We are unaware of a prior genetic study where ascertainment was primarily by ocular examination. There were two children in our cohort with the rare form of Stickler syndrome from autosomal recessive collagen type IX disease (subject #3 and 4, both *COL9A1*‐related). The vitreous phenotype in these children was nonspecific, consistent with what has been reported in the literature (Nixon et al., 2019). To date, 10 other families with collagen type IX‐related Stickler syndrome have been reported (Nixon et al., 2019). All affected individuals had myopia and hearing impairment and many had joint pain. Only 5% had retinal detachment at the time of assessment (as opposed to 50% or more in autosomal dominant series (Rose et al., 2005; Spallone, 1987)); however, the incidence of retinal detachment likely increased with time. Recessive collagenopathy accounted for only 2/10 collagenopathy patients in this series despite the fact that recessive forms of ocular genetic disease are relatively common in the Middle East region (Khan, 2013). Again, this is likely related to our method of patient ascertainment, which selected against patients with obvious syndromic disease who would have been more likely to be already diagnosed. Recessive phenotypes for what is classically considered autosomal dominant disease is a phenomenon in the region (Khan, 2013). Recessive phenotypes typically (although not always) have obvious and more severe multisystem disease (Khan et al., 2014; Monies et al., 2017). An example of a recessive *COL11A1* phenotype is provided in Figure S1.

Two children in this series did not have detectable collagen gene mutations by our methodology. One remains idiopathic (subject #12). The other was found to have *LRPAP1*‐related very high myopia (subject #11). In addition to extreme myopia and vitreous changes, he also had flat facies, which increased suspicion for Stickler syndrome. In retrospect, his nonspecific vitreous changes were related to his very high myopia rather than primary vitreous disease and his flat facies were an unrelated finding. Such extreme myopia in a young child from this region of the world should raise suspicion for mutations in *LRPAP1*, but the time he was assessed and included in this study was before we had a clear understanding of the features that suggest *LRPAP1*‐related extreme myopia (Khan et al., 2016). This child's case highlights the utility of molecular genetic diagnosis for clinical refinement and proper counseling. Accompanying signs such as flat facies that are suggestive for a syndromic diagnosis can sometimes be misleading coincidence (Allanson et al., 2009; Khan, 2017).

## CONFLICT OF INTEREST

No author has a conflict of interest to disclose.

## AUTHOR CONTRIBUTIONS

Have made substantial contributions to conception and design, or acquisition of data, or analysis and interpretation of data: Arif O. Khan, Lama AlAbdi, Nisha Patel, Rana Helaby, Mais Hashem, Firdous Abdulwahab, Fahad B. AlBadr, Fowzan S. Alkuraya. Been involved in drafting the manuscript or revising it critically for important intellectual content: Arif O. Khan, Lama AlAbdi, Nisha Patel, Rana Helaby, Mais Hashem, Firdous Abdulwahab, Fahad B. AlBadr, Fowzan S. Alkuraya. Given final approval of the version to be published. Each author should have participated sufficiently in the work to take public responsibility for appropriate portions of the content: Arif O. Khan, Lama AlAbdi, Nisha Patel, Rana Helaby, Mais Hashem, Firdous Abdulwahab, Fahad B. AlBadr, Fowzan S. Alkuraya. Agreed to be accountable for all aspects of the work in ensuring that questions related to the accuracy or integrity of any part of the work are appropriately investigated and resolved: Arif O. Khan, Lama AlAbdi, Nisha Patel, Rana Helaby, Mais Hashem, Firdous Abdulwahab, Fahad B. AlBadr, Fowzan S. Alkuraya.

## Supporting information

Fig S1

Table S1

## Data Availability

The data sets generated during and/or analyzed during the current study are available from the corresponding author on reasonable request.
