# Supplementary material for: Genetic testing results of children suspected to have Stickler syndrome type collagenopathy after ocular examination
Source: Mol Genet Genomic Med. 2021 May 5;9(5):e1628. doi: 10.1002/mgg3.1628 (PMC8172201; doi:10.1002/mgg3.1628)
Supplement: Supplementary file 1 — Fig S1 [file MGG3-9-e1628-s002.pdf]

Sibling 1

A 17-year-old boy had midface hypoplasia, depressed nasal bridge, small nose, joint abnormalities, and short stature. He also had developmental delay and delayed speech.

Evaluation revealed expressive language delay. Brain auditory evoked potential was normal except for increased hearing threshold. He had severe myopia. Parents were related and he has a brother with similar findings (Sibling 2).

Sibling 2

A 10-year-old boy had midface hypoplasia, depressed nasal bridge, small nose, bilateral hearing loss, subluxation of the lenses, esotropia, high myopia, right retinal detachment, and joint abnormalities. He also had developmental delay and delayed speech.

A

Sibling 1

Sibling 2

Both siblings

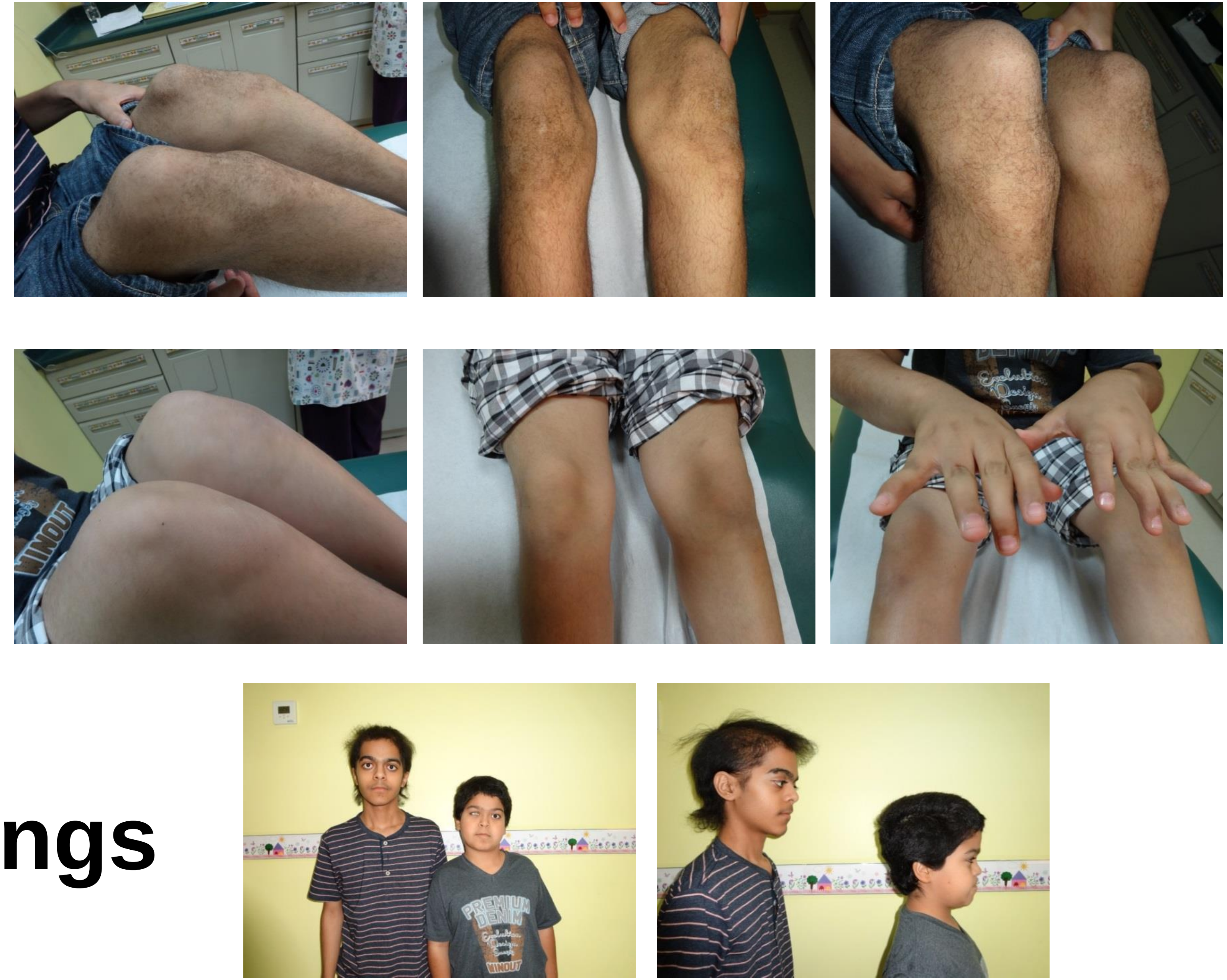

B

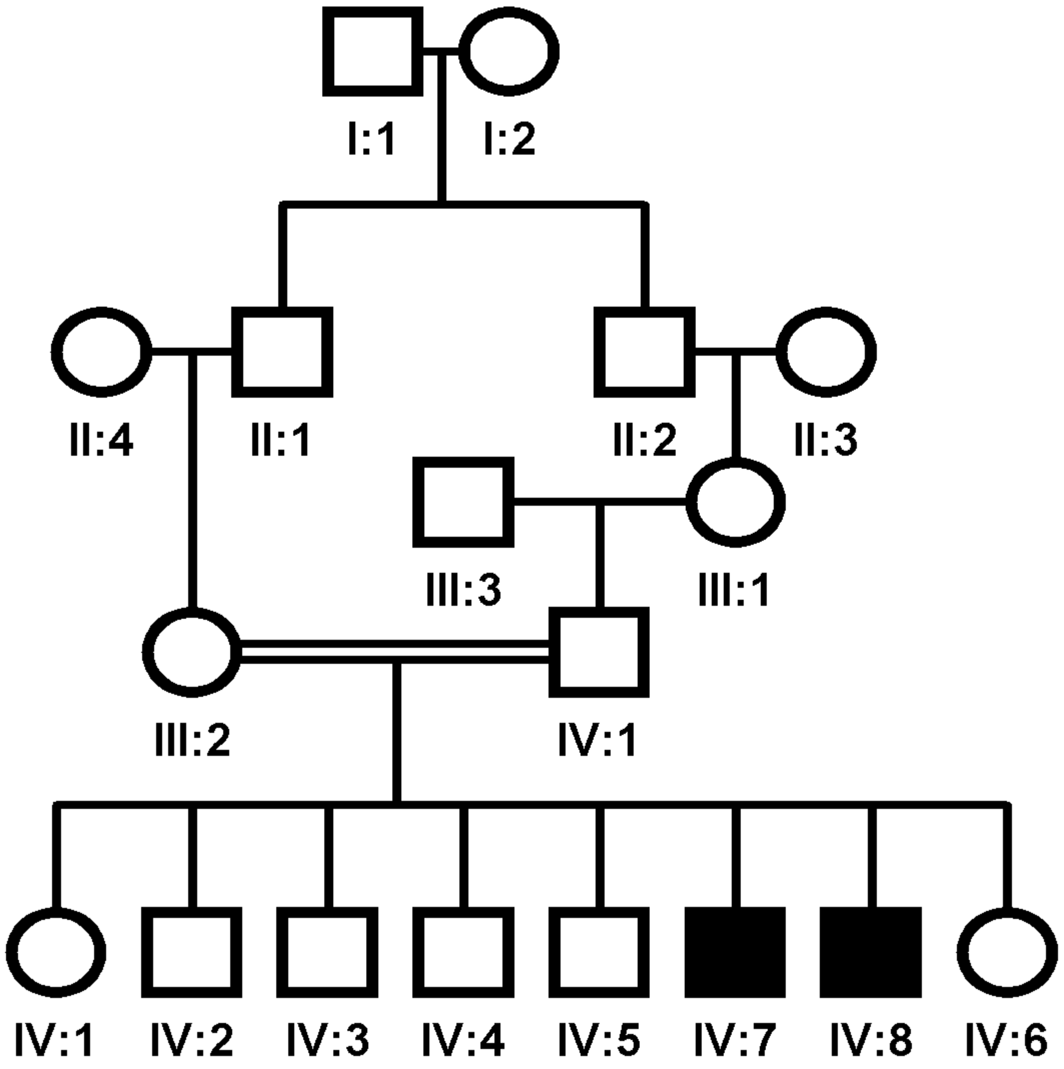

COL11A1: NM_001854.3:c.2702G>A; p.(Gly901Glu) [Homozygous]

LEGEND FOR E-FIGURE:

E-Figure 1: An example of a recessive *COL11A1* phenotype: (A) Clinical photographs showing joint abnormalities (Sibling 1 and 2). Bottom panel highlights facial dysmorphology such as midface hypoplasia, depressed nasal bridge, small nose (right eye retinal detachment of sibling 2 on the right). (B) Pedigree showing consanguinity between the parents of the affected siblings.
