# Supplementary material for: Genetic testing results of children suspected to have Stickler syndrome type collagenopathy after ocular examination
Source: Mol Genet Genomic Med. 2021 May 5;9(5):e1628. doi: 10.1002/mgg3.1628 (PMC8172201; doi:10.1002/mgg3.1628)
Supplement: Supplementary file 2 — Table S1 [file MGG3-9-e1628-s001.pdf]

Gene	HGVS nomenclature	Zygosity	ACMG criteria points	ACMG classification	OMIM phenotype related to gene	Previously reported	Additional remarks	Subjects # with this mutation
COL2A1	NM_001844.5:c.2659C>T; p.(Arg887*)	Het	PVS1, PM1, PM2, PP3, and PP5	Pathogenic	1. Stickler syndrome type I [AD]<br>2. Stickler sydrome, type I, nonsyndromic ocular [AD]<br>3. Achondrogenesis, type II or hypochondrogenesis [AD]<br>4. Epiphyseal dysplasia, multiple, with myopia and deafness [AD]<br>5. Kniest dysplasia [AD]<br>6. Legg-Calve-Perthes disease [AD]<br>7. Osteoarthritis with mild chondrodysplasia [AD]<br>8. Platyspondylic skeletal dysplasia, Torrance type [AD]<br>9. SED congenita [AD]<br>10. SMED Strudwick type [AD]<br>11. Spondyloepiphyseal dysplasia, Stanescu type [AD]<br>12. Spondyloperipheral dysplasia [AD]<br>13. Czech dysplasia [AD]<br>14. Avascular necrosis of the femoral head [AD]<br>15. Vitreoretinopathy with phalangeal epiphyseal dysplasia	PMID: 16752401	Lies within a critical 'Triple-helical region' where 85.3% of variants are pathogenic. HGMD accession number (CM062564)	#1
COL2A1	NM_001844.5:c.2818C>T; p.(Arg940*)	Het	PVS1, PM1, PM2, PP3, and PP5	Pathogenic	1. Stickler syndrome type I [AD]<br>2. Stickler sydrome, type I, nonsyndromic ocular [AD]<br>3. Achondrogenesis, type II or hypochondrogenesis [AD]<br>4. Epiphyseal dysplasia, multiple, with myopia and deafness [AD]<br>5. Kniest dysplasia [AD]<br>6. Legg-Calve-Perthes disease [AD]<br>7. Osteoarthritis with mild chondrodysplasia [AD]<br>8. Platyspondylic skeletal dysplasia, Torrance type [AD]<br>9. SED congenita [AD]<br>10. SMED Strudwick type [AD]<br>11. Spondyloepiphyseal dysplasia, Stanescu type [AD]<br>12. Spondyloperipheral dysplasia [AD]<br>13. Czech dysplasia [AD]<br>14. Avascular necrosis of the femoral head [AD]<br>15. Vitreoretinopathy with phalangeal epiphyseal dysplasia	PMID: 27408751; ClinVar submission (RCV000438911)	Lies within a critical hotspot of 61 base-pairs with all 11 variants described are pathogenic; persumed *de novo* as parents are unrelated	#2
COL9A1	NM_001851.4:c.1052C>A; p.(Ser351*)	Homo	PVS1, PM2, PP3, and PP5	Pathogenic	1. Stickler syndrome type IV [AR]	ClinVar (RCV000733131)		#3
COL9A1	NM_001851.4:c.2068_2069del; p.(Arg690Glyfs*17)	Homo	PVS1, PM1, PM2, and PP3	Pathogenic	1. Stickler syndrome type IV [AR]		Lies within a critical 'Triple-helical region' where 31.8% of variants reported are pathogenic	#4
COL11A1	NM_001854.3:c.3756_3762del; p.(Glu1253ValfsTer17)	Het	PVS1, PM1, PM2, and PP3	Pathogenic	1. Stickler syndrome, type II [AD]<br>2. Fibrochondrogenesis 1 [AR]<br>3. Marshall syndrome [AD]		Lies within a 'Triple-helical region' where 58.9% of variants reported are pathogenic; segregates within the family	#5
COL11A1	NM_001854.3:c.1945-1G>C	Het	PVS1, PM2, and PP3	Pathogenic	1. Stickler syndrome, type II [AD]<br>2. Fibrochondrogenesis 1 [AR]<br>3. Marshall syndrome [AD]		*in silico:* TraP: 0.577	#6
COL11A1	NM_001854.3:c.2241+5G>T	Het	PM2 and BP4	VOUS	1. Stickler syndrome, type II [AD]<br>2. Fibrochondrogenesis 1 [AR]<br>3. Marshall syndrome [AD]		*in silico:* TraP: 0.957	#7
COL11A1	NM_001854.3:c.4412G>A; p.(p.Gly1471Asp)	Het	PM1, PM2, PP2, and PP3	Likely Pathogenic	1. Stickler syndrome, type II [AD]<br>2. Fibrochondrogenesis 1 [AR]<br>3. Marshall syndrome [AD]	PMID: 17236192	in silico: pathogenic by 11 prediction tools DANN, DEOGEN2, EIGEN, FATHMM-MKL, M-CAP, MVP, MutationAssessor, MutationTaster, PrimateAI, REVEL and SIFT. HGMD accession number (CM070056)	#8, 9
COL11A1	NM_001854.3:c.3816+1G>A	Het	PVS1, PM2, PP3 and PP5	Pathogenic	1. Stickler syndrome, type II [AD]<br>2. Fibrochondrogenesis 1 [AR]<br>3. Marshall syndrome [AD]	PMIDs: 25240749, 21668896, 21035103, 19449424, 17236192, 13520885, 9792885, 9529347, 9129742 ; ClinVar: RCV000579344, RCV000623510, RCV000032995	*in silico:* TraP: 0.937. HGMD accession number (CS982120)	#10
LRPAP1	NM_002337.3:c.863_864del; p.(Ile288Argfs*118)	Homo	PVS1, PM2, PP3 and PP5	Pathogenic	1. Myopia 23 [AR]	PMID: 23830514		#11
